# Diversity of Endodontic *Enterococcus faecalis* from Brazil with a High Occurrence of the ST 397 Clone

**DOI:** 10.1590/0103-644020246104

**Published:** 2024-12-16

**Authors:** Renata Ximenes Lins, Fernanda Santos Freitas, Ana Carolina Paulo Vicente

**Affiliations:** 1 Federal Fluminense University, Post-Graduation Program in Dentistry, Healthy Institute of Nova Friburgo, Nova Friburgo, Brazil; 2 Instituto Oswaldo Cruz, Laboratório de Genética Molecular de Microrganismos, FIOCRUZ, Rio de Janeiro, Brazil

**Keywords:** Enterococcus faecalis, genotypes, MLST, root canals, antimicrobial resistance

## Abstract

*Enterococcus faecalis* is a common cause of nosocomial infections and is frequently associated with endodontic infections. However, there is a scarcity of studies addressing the genetic characterization of *E. faecalis* lineages most commonly associated with root canals. The aim of this study was to assess the genetic relatedness of *E. faecalis* strains from primary endodontic infections in Southeast Brazil, test the hypothesis of clones infecting unrelated individuals, and examine the antimicrobial resistance profile. The genetic relationship of 32 endodontic *E. faecalis* isolates was investigated using multilocus sequence typing (MLST). These isolates were collected from unrelated patients with primary endodontic infections treated in Brazil between 2010 and 2023. Antimicrobial susceptibility testing was performed using the disk diffusion method in accordance with the Clinical Laboratory Standards Institute guidelines. Twelve sequence types (STs) were detected, of which eight STs contained only a single strain. Clones of ST 30, ST 40, ST 97, and ST 397 were identified, with a notably high frequency of ST 397 (15/32). Susceptibility to the antimicrobial agents tested varied, with the highest resistance rates observed for clindamycin (100%), tetracycline (34.4%), azithromycin (31.2%), and ciprofloxacin (19.2%). One isolate was found to be multidrug-resistant. MLST analysis of endodontic *E. faecalis* revealed clones infecting different individuals in various cities over a span of up to 10 years, with a high occurrence of ST 397. Therefore, there appears to be a predominant *E. faecalis* lineage associated with endodontic infections in Southeast Brazil. These findings, together with existing literature, raise concerns that certain lineages may be specialized in causing endodontic infections.

## Resumo

*Enterococcus faecalis*, causa comum de infecções nosocomiais, é frequentemente associado a infecções endodônticas. Entretanto, há poucos estudos de caracterização genética das linhagens mais associadas aos canais radiculares. O objetivo deste estudo foi avaliar a relação genética entre isolados de *E. faecalis* de infecções endodônticas primárias no sudeste do Brasil para testar a hipótese de clones infectando indivíduos não relacionados e examinar o perfil de resistência antimicrobiana. A relação genética de 32 *E. faecalis* endodônticos foi obtida pela técnica de tipagem de sequências multilocus (MLST). Esses isolados foram coletados de pacientes não relacionados com infeção endodôntica primária tratados no Brasil entre 2010 e 2023. Teste de suscetibilidade antimicrobiana foi realizado utilizando a técnica de difusão em disco de acordo com o *Clinical Laboratory Standard Institute* (CLSI). Foram detectados 12 tipos de sequência (STs), dos quais 8 STs continham apenas um isolado. Clones de ST 30, ST 40, ST 97 e ST 397 foram encontrados, com uma elevada frequência deste último (15/32). A suscetibilidade aos agentes antimicrobianos testados variou, com a maior percentagem de resistência observada para a clindamicina (100%), tetraciclina (34,4%), azitromicina (31,2%) e ciprofloxacina (19,2%). Um isolado era multirresistente. O MLST de *E. faecalis* endodônticos revelou clones infectando diferentes indivíduos em diferentes cidades, com até 10 anos de intervalo, com alta ocorrência do ST 397. Portanto, existe uma linhagem predominante de *E. faecalis* associada a infecções endodônticas no sudeste do Brasil. Estes dados, juntamente com a literatura, levantam a preocupação de que possam existir linhagens especializadas em infecções endodônticas.

## Introduction

*Enterococcus faecalis*, a facultative aerobic Gram-positive coccus, is considered the major pathogen responsible for persistent endodontic infections following root canal treatment and is the most extensively studied bacterium in endodontic microbiology research ^1^. Its ubiquity and genetic plasticity allow it to survive under adverse conditions, such as nutrient scarcity and low oxygen tension within root canals ^2^, as well as desiccation, starvation, and disinfection in modern hospital environments, predicting its emergence as a leading hospital pathogen ^3^.

While hundreds of scientific articles have been published on endodontic infections and *E. faecalis*, the vast majority have focused on testing antimicrobial methods against this microorganism. Although the association between enterococci and root canal infections is well established, few studies have investigated their origin, genetic relationships, and whether specific lineages are more commonly associated with these infections ^4^ ^,^ ^5^ ^,^ ^6^ ^,^ ^7^ ^,^ ^8^ ^,^ ^9^ ^,^ ^10^. Identifying clones specialized in causing endodontic infections could lead to more effective therapeutic strategies.

Recently, Gaeta et al. ^8^ investigated the phylogenetic relationship of *E. faecalis* isolates from root canals and saliva, revealing two genetically related isolates from the root canals of different patients. Vidana et al. ^9^ examined the origin of endodontic isolates by comparing the genetic profile of virulence and antimicrobial resistance of blood and food isolates. They concluded that, although it was possible to detect common genotypic characteristics between strains by comparing genetic patterns, a clearer association may have been masked by the extraordinary plasticity of the *E. faecalis* genome. They emphasized the need for methods such as multilocus sequence typing (MLST) in future epidemiologic analyses. Based on the sequence analysis of housekeeping genes, MLST is the gold standard tool for bacterial typing and clustering analysis. It is a powerful technique for addressing relatedness within species, and it allows for comparisons with a global open-access database (https://pubmlst.org/organisms/enterococcus-faecalis). To the best of our knowledge, Pinheiro et al. ^6^ ^,^ ^10^ were the first and only group to have used MLST to study the relatedness of endodontic E. faecalis lineages.

Another concern regarding *E. faecalis* is its antimicrobial resistance. Recently, Ardilla et al. ^11^ reviewed antimicrobial resistance in endodontic infections, and Barbosa-Ribeiro et al. ^12^ focused on endodontic enterococci. Both reports expressed alarm at the increasing antimicrobial resistance of endodontic *E. faecalis* over time. The ease with which enterococci can acquire new antimicrobial resistance genes, including through the transfer of PAIs, is well recognized and underscores the need for continuous monitoring of clinical isolates from different sites of infection and geographical origins ^3^.

Therefore, the objective of this study was to evaluate the genetic relatedness of *E. faecalis* strains from primary endodontic infections in Southeast Brazil using MLST, to test the hypothesis of the existence of clones infecting unrelated individuals, and to examine the antimicrobial resistance profile of these isolates.

## Materials and Methods

### Sample collection

A collection of 32 *E. faecalis* isolates was retrospectively evaluated. These isolates were collected from unrelated patients with primary endodontic infection treated in Brazil between 2010 and 2023 accordingly previously described by Lins et al. ^7^. Only necrotic pulps without oral exposure were included. All patients gave written informed consent, were otherwise healthy, and had not been hospitalized or received antibiotics for at least 6 months prior to the study. In 2010, the samples were collected in the city of Rio de Janeiro (RJ), while in 2020 and 2023 they were collected in the city of Nova Friburgo (NF). The latest isolates were obtained in a study approved by the Research Ethics Committee of the Federal University of Fluminense (CAAE 87647218.6.0000.5626).

### E. faecalis identification by sequencing of 16S ribosomal DNA

Total DNA was extracted using the PureLink™ Genomic DNA Mini Kit (Invitrogen, Brazil) according to the manufacturer's recommended protocol. The extracted DNA was used as a template for PCR using the universal bacterial 16S rRNA primer pair of 27f and 1492r ^7^.

### Antimicrobial susceptibility testing (AST)

Antimicrobial susceptibility testing was performed using the disk diffusion technique on Müller-Hinton agar (Merk, Germany) according to the Clinical Laboratory Standard Institute (CLSI) recommendations (CLSI M100, 2023) ^13^. Using a sterile wire loop, 3-5 pure colonies were transferred to a tube containing 5 mL of sterile normal saline (0.85% NaCl) and gently mixed until a uniform suspension was obtained. The standard inoculum density was adjusted to 0.5 McFarland units. The bacterial suspension was swabbed onto the MHA surface using a sterile swab, then a set of antibiotic discs were placed with sterile forceps at least 24 mm apart. Susceptibility was tested using antibiotic discs (Oxoid, England) against Erythromycin (15 µg), Azithromycin, Ampicillin (10 µg), Penicillin (10 units), Amoxicillin + Clavulanate (3 µg), Ciprofloxacin (5 µg), Clindamycin (30 µg), Tetracycline (30 µg), Vancomycin (30 µg). The plates were then incubated at 37 °C for 18-24 h. Each zone of inhibition was measured to the nearest millimeter and classified as sensitive, intermediate or resistant using the standard technique ^(^ ^13^. Although azithromycin is not included in the *Enterococcus* resistance classification due to the lack of species-specific breakpoints, we still analyzed it as it is one of the most prescribed drugs for endodontic infections, particularly in cases of penicillin allergy. The results were interpreted according to the CLSI ^13^, and in the case of azithromycin, the breakpoints for*Enterobacteriaceae*(susceptible ≥16 mm; intermediate11-15 mm, and resistant ≤10 mm) were applied.

### Genetic Relatedness

As described by Ruiz-Garbajosa et al. ^14^, multilocus sequence typing (MLST) was used to characterize clones and analyze genetic relationships. Alleles and sequence types (STs) were assigned using the global MLST database for *Enterococcus faecalis*, available at https://pubmlst.org/organisms/enterococcus-faecalis. The study evaluated seven genes: *gdh* for glucose-6-phosphate dehydrogenase, *gyd* for glyceraldehyde-3-phosphate dehydrogenase, *pstS* for phosphate-adenosine triphosphate-binding cassette transporter, *gki* for putative glucokinase, *aroE* for shikimate-5 dehydrogenase, *xpt* for shikimate-5 dehydrogenase, and *yiqL* for acetyl-coenzyme A acetyltransferase. PCR conditions for all amplification reactions were as follows: initial denaturation at 94 C for 5 min; 30 cycles at 94 C for 30 s, 52 C for 30 s, and 72 C for 1 min; and final extension at 72 C for 10 min. The amplicons were purified using the PureLink Quick PCR Purification Kit (Invitrogen, Brazil), following the manufacturer's instructions. The fragments were then Sanger sequenced using the BigDye Terminator Cycle Sequencing Ready Reaction Kit (Applied Biosystems, USA). For each isolate, each gene was amplified and sequenced using the specific forward or reverse primer. For each locus, a unique allele number was assigned to each different sequence according to the *E. faecalis* MLST database. We investigated the relatedness between the different STs and constructed a phylogenetic tree using Molecular Evolutionary Genetic Analysis (MEGA-X). The highly aligned sequence in FASTA format was used to build a model in the phylogenetic tree, each of the branches was labeled with the *E. faecalis* isolate number along with the year of isolation.

## Results

### Antimicrobial susceptibility testing (AST)

Antimicrobial susceptibility of *E. faecalis* varied and is shown in Table 1. All strains were susceptible to ampicillin, amoxicillin with clavulanate and vancomycin. Susceptibility to the other antimicrobial agents tested varied, with the highest percentage of resistance observed for clindamycin (100%), tetracycline (34.4%), azithromycin (31.2%) and ciprofloxacin (19.2%). Intermediate susceptibility to erythromycin was identified in 31 isolates (96.8%), to ciprofloxacin in 26 isolates (81.2%), to azithromycin in 20 isolates (62.5%). One isolate, Ef. 8, was the only one resistant to penicillin and found to be multidrug resistant.

### Genetic Relatedness

**Figure 1:**
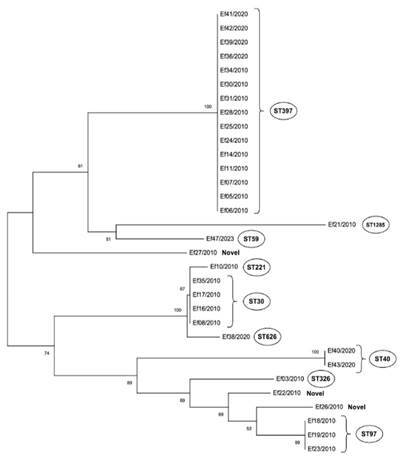
Phylogenetic tree of *E. faecalis* constructed by MEGA X using Neighbor-Joining method.

MLST of the 32 isolates revealed 12 sequence types (STs), of which 8 STs comprised only one isolate (Figure 1). The ST 397 was the most frequent (15/32), representing 48% of the studied group. Another four isolates were assigned into ST 30, three into ST 97, two in ST 40 and five were resolved into different STs with one isolate each: 59, 221, 326, 626 and 1285. *E. faecalis* from these STs were found in hospitalized patients, human community, animal and food in Europe, North America and Latin America countries. However, in our collection there are 3 new STs.

The ST 397 strains found in our study were isolated in 2010 and 2020 in different cities, Rio de Janeiro and Nova Friburgo, respectively. These results reveal the presence of a *E. Faecalis* clone persisting for a decade in endodontic infection in different cities of Rio de Janeiro state. Other clones from ST 30 and ST 97, 2010, were also revealed associated to endodontic infections in Rio de Janeiro, while clones from ST 40, 2020, in Nova Friburgo.

**Table 1:** Antimicrobial susceptibility of *E. faecalis*. Resistance (R), Intermediate (I) and susceptibility (S).

Ef n.	PEN	AMP	AMC	ERY	AZI	TET	CLI	CIP	VAN
3	S	S	S	I	I	I	R	R	S
5	S	S	S	I	S	S	R	R	S
6	S	S	S	I	R	S	R	I	S
7	S	S	S	I	R	S	R	I	S
8	R	S	S	R	I	R	R	R	S
10	S	S	S	I	I	R	R	I	S
11	S	S	S	I	R	S	R	I	S
14	S	S	S	I	I	R	R	I	S
16	S	S	S	I	R	R	R	I	S
17	S	S	S	I	I	R	R	I	S
18	S	S	S	I	I	R	R	I	S
19	S	S	S	I	I	R	R	I	S
21	S	S	S	I	I	I	R	R	S
22	S	S	S	I	S	R	R	I	S
23	S	S	S	I	I	S	R	I	S
24	S	S	S	I	R	S	R	I	S
25	S	S	S	I	R	S	R	I	S
26	S	S	S	I	I	S	R	I	S
27	S	S	S	I	I	S	R	I	S
28	S	S	S	I	I	S	R	I	S
30	S	S	S	I	I	S	R	I	S
31	S	S	S	I	I	S	R	R	S
34	S	S	S	I	I	R	R	I	S
35	S	S	S	I	R	R	R	I	S
36	S	S	S	I	I	S	R	I	S
38	S	S	S	I	R	S	R	I	S
39	S	S	S	I	I	S	R	I	S
40	S	S	S	I	R	R	R	I	S
41	S	S	S	I	I	S	R	I	S
42	S	S	S	I	R	S	R	I	S
43	S	S	S	I	I	S	R	I	S
47	S	S	S	I	I	S	R	R	S

## Discussion

This study characterized the diversity of endodontic *E. faecalis* across different times and locations in the state of Rio de Janeiro, Brazil, and identified *E. faecalis* lineages associated with these infections. Notably, it revealed a high occurrence (15/32) of ST 397 in unrelated individuals from different cities within the state, up to 200 km apart and spanning a decade. In addition, clones of ST 30, ST 40, and ST 97 were also identified, supporting the hypothesis that specific *E. faecalis* lineages are associated with endodontic infections.

Using the MLST approach, Sun et al. ^5^ identified clones of *E. faecalis* in marginal periodontitis in Norway. Their study was the first to apply MLST to a large collection of oral *E. faecalis* isolates from marginal periodontitis and root canals. They reported 40 STs, of which 23 were unique to a single isolate, and identified clones of ST 21, ST 40, ST 30, and ST 56. Pinheiro et al. ^12^ also identified clones belonging to ST 25, ST 62, ST 79, and ST 396 in endodontic *E. faecalis*, with 42.8% assigned to the same clonal complex (CC25), which includes ST 97, also found in this study (n=3). These studies highlight the importance of MLST in tracking the epidemiology of endodontic *E. faecalis*.4

Despite the limited sample size in this study, observations on the epidemiology of endodontic infections by *E. faecalis* were made possible by accessing the global MLST database. In Brazil, 146 isolates have been recorded, resulting in 75 different STs. Given these figures, the high occurrence (46.8%) of the same ST (397) in a regional collection of 32 isolates supports the hypothesis that certain clonal groups may be more specialized in endodontic infections.

Obtaining a large collection of endodontic *E. faecalis* isolates is challenging because root canal sampling is a complex procedure ^7^, and the microorganism is not always successfully isolated. In the study by Sun et al. ^5^, while 108 isolates were obtained from marginal periodontitis, only two were from endodontic infections. These two endodontic strains were isolated from the root canals of 21 patients in Norway between 2005 and 2006. Interestingly, one of them belonged to ST 40, which was also found in endodontic infections in Brazil, isolated in 2003 ^15^ and again in 2020 (this study). This finding indicates the existence of a lineage capable of infecting root canals, circulating in both Norway and Brazil, and persisting over several years. As of January 2024, the global database contains 1,857 annotated STs of *E. faecalis*, with 75 from Brazil. ST 40 has been documented 73 times in the database, having been isolated worldwide from various human, food, and animal sources, ranging from hospital to community settings. The ability to persist across different ecosystems has led enterococci to become one of the indicator bacteria for monitoring the transmission of virulence factors from a One Health perspective ^16^, making lineage definition crucial for tracking.

Regarding ST 397, it has only been reported in Brazil and was the predominant ST associated with our isolates in both sampling years (2010 and 2020), demonstrating its persistence over the years in endodontic infections in Southeast Brazil. The first recorded case of ST 397 in a patient with an endodontic infection was in São Paulo in 2003 ^12^ ^,^ ^15^. This suggests that ST 397 has been present in the southeastern region of Brazil for at least 17 years and may have the potential to infect root canals of unrelated patients. Another study that also isolated ST 397 in Brazil was conducted by Santos et al. ^17^, who analyzed the genetic relationships among clinical *E. faecalis* isolates (n = 26) obtained from cancer patients in the state of Rio de Janeiro.

Notably, lineages associated with endodontic infections can circulate in hospitals. In addition to ST 397, Santos et al. ^17^ identified strains from ST 626 and ST 59, both of which exhibited multidrug-resistant profiles. Although these STs were present in the collection of this study, they did not exhibit the same level of multidrug resistance (see Table 1). Furthermore, Penas et al. ^6^ reported ST 30, also present in this collection, in intensive care patients in Brazil and in clinical strains from Europe, Asia, Africa, and Oceania, primarily in hospitalized patients. Their study also showed that *E. faecalis* from clinical infections harbored more virulence factors, whereas those from endodontic infections were more diverse and contained fewer virulence and antibiotic resistance genes.

MLST, based on the sequencing of a set of housekeeping genes, determines the genetic relationship between isolates, and the sequence type (ST) information can be used to access other local and global collections, helping establish the epidemiology of the bacteria. However, most studies in endodontic microbiology have employed genetic methods such as PFGE and RAPD, which do not provide comparability. The main advantage of MLST over other typing methods is the unambiguous and transferable nature of the sequence data ^19^. In clinical practice, defining lineages allows for better treatment follow-up and monitoring of the antibiotic resistance profile of each lineage, as well as the investigation of more specific therapies against the most prevalent and virulent clones.

According to Barbosa-Ribeiro et al. ^12^, there has been an increase in the resistance profile of *E. faecalis* to vancomycin, chloramphenicol, gentamicin, metronidazole, moxifloxacin, and tigecycline in recent years. However, resistance to vancomycin was not observed in this study. A systematic review by Ardilla et al. ^11^ found no reports of vancomycin-resistant *E. faecalis* from root canals.

In this study, MLST revealed diversity among endodontic *E. faecalis* isolates, as well as clones infecting different individuals from various cities up to 200 km apart and a decade apart.

This raises concerns that there may be clones specialized in endodontic infections.
